# The role of the microbiota-gut-brain axis and intestinal microbiome dysregulation in Parkinson’s disease

**DOI:** 10.3389/fneur.2023.1185375

**Published:** 2023-05-25

**Authors:** Qing Li, Ling-bing Meng, Li-jun Chen, Xia Shi, Ling Tu, Qi Zhou, Jin-long Yu, Xin Liao, Yuan Zeng, Qiao-ying Yuan

**Affiliations:** ^1^Department of Nutrition, Southwest Hospital, Third Military Medical University (Army Medical University), The First Affiliated Hospital of PLA Army Medical University, Chongqing, China; ^2^Department of Cardiology, Beijing Hospital, National Center of Gerontology, Institute of Geriatric Medicine, Chinese Academy of Medical Sciences, Beijing, China

**Keywords:** Parkinson’s disease, microbiota-gut-brain axis, intestinal microbiome dysregulation, inflammation, gastrointestinal dysfunction

## Abstract

Parkinson’s disease (PD) is a complex progressive neurodegenerative disease associated with aging. Its main pathological feature is the degeneration and loss of dopaminergic neurons related to the misfolding and aggregation of α-synuclein. The pathogenesis of PD has not yet been fully elucidated, and its occurrence and development process are closely related to the microbiota-gut-brain axis. Dysregulation of intestinal microbiota may promote the damage of the intestinal epithelial barrier, intestinal inflammation, and the upward diffusion of phosphorylated α-synuclein from the enteric nervous system (ENS) to the brain in susceptible individuals and further lead to gastrointestinal dysfunction, neuroinflammation, and neurodegeneration of the central nervous system (CNS) through the disordered microbiota-gut-brain axis. The present review aimed to summarize recent advancements in studies focusing on the role of the microbiota-gut-brain axis in the pathogenesis of PD, especially the mechanism of intestinal microbiome dysregulation, intestinal inflammation, and gastrointestinal dysfunction in PD. Maintaining or restoring homeostasis in the gut microenvironment by targeting the gut microbiome may provide future direction for the development of new biomarkers for early diagnosis of PD and therapeutic strategies to slow disease progression.

## 1. Introduction

**Figure 1:**
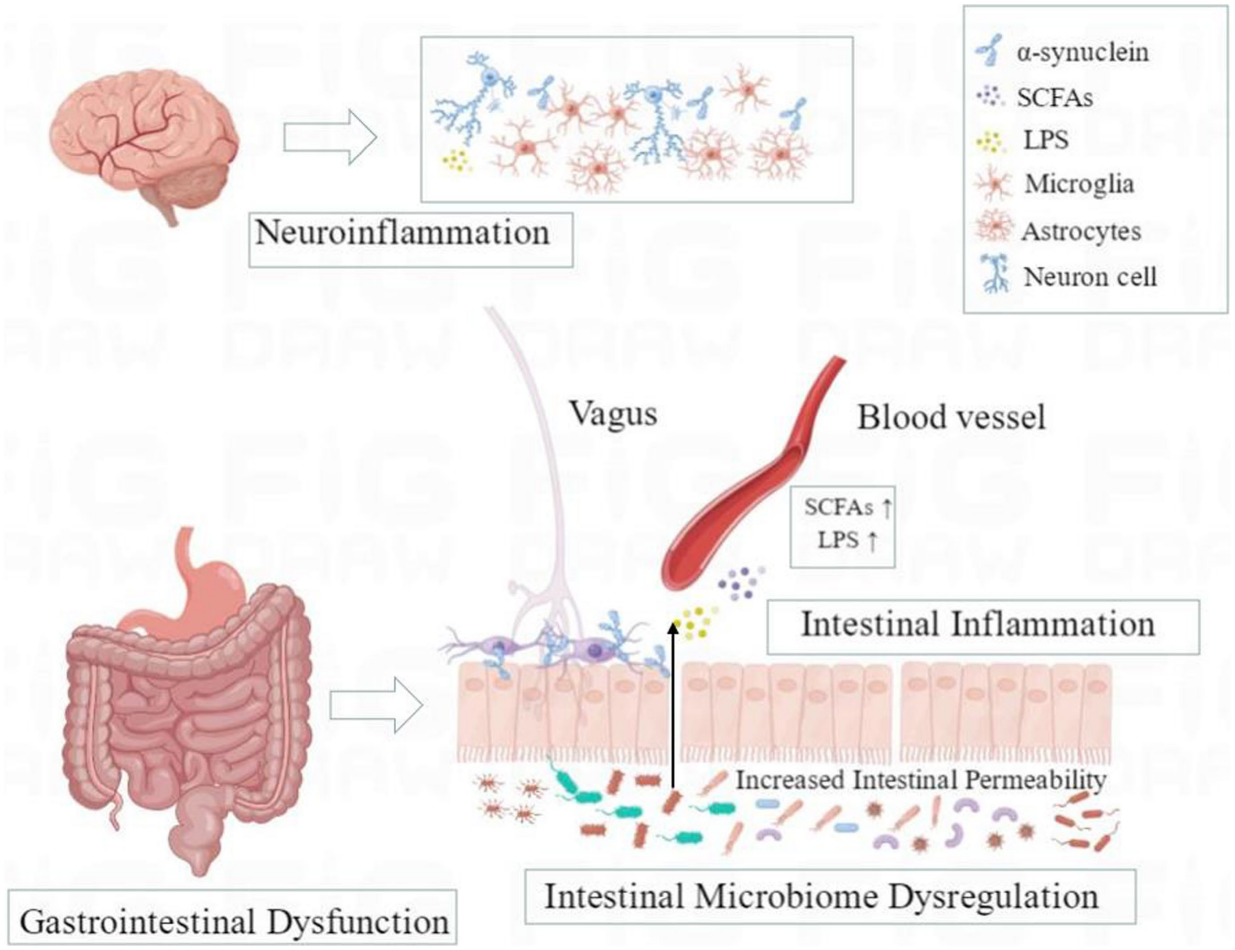
A schematic overview of microbiota-gut-brain axis in Parkinson’s disease. Dysregulation of intestinal microbiota may promote the damage of the intestinal epithelial barrier, intestinal inflammation, and the upward diffusion of phosphorylated α-synuclein from the enteric nervous system (ENS) to the brain through the vagus nerve in susceptible individuals and further lead to gastrointestinal dysfunction, neuroinflammation, and neurodegeneration of the central nervous system (CNS) through the disordered microbiota-gut-brain axis.

Parkinson’s disease (PD) is a progressive neurodegenerative disease associated with aging (1), and its prodromal stage may be longer than 20 years. The prodromal stage is characterized by specific non-motor symptoms, including rapid eye movement sleep behavior disorder (RBD), autonomic nerve dysfunction, and cognitive disorders (1, 2). The main pathological feature of PD is the progressive loss of dopaminergic neuron (DN), which is related to the misfolding and aggregation of α-synuclein (1–3). However, α-synuclein could be detected in both central nervous system (CNS) and enteric nervous system (ENS). Studies on animal models of PD indicate that abnormal α-synuclein may spread to the CNS in a prion-like manner through the vagus (4, 5). In the pathogenesis and development of PD, the intestinal microbiome affects the close two-way communication between the gastrointestinal tract and the brain, which is called the microbiota-gut-brain axis (Figure 1). PD patients have significant intestinal microbiome disorders and metabolite changes, which may promote the damage of the intestinal epithelial barrier, intestinal inflammation, and abnormal phosphorylation of α-synuclein to spread upward from the ENS to the brain in individuals with genetic susceptibility and further lead to gastrointestinal dysfunction, neuroinflammation, and neurodegeneration of CNS through the disordered microbiota-gut-brain axis (6, 7). Currently, no treatment can cure or effectively prevent the progression of PD. Although dopamine replacement therapy helps to improve the initial motor symptoms, it cannot inhibit dopaminergic neurodegeneration and is associated with motor complications (8, 9). Meanwhile, oral administration of levodopa and other PD-related drugs requires the optimal gastrointestinal function to determine the ideal drug metabolism. However, gastrointestinal dysfunction and intestinal microbiome disorders in PD patients will interfere with the absorption and utilization of drugs (10–15), while some therapeutic agents (such as dopamine agonists) may directly affect the gut microbiome and aggravate gastrointestinal dysfunction (16–18). Therefore, there is an urgent need to better determine the pathobiological mechanism of the highly complex bidirectional association of the microbiota-gut-brain axis in PD. Hence, in this review, we aimed to summarize recent advancements in studies focusing on the role of the microbiota-gut-brain axis in the pathogenesis of PD, especially the potential mechanism of intestinal microbiome dysregulation, intestinal inflammation, and gastrointestinal dysfunction in PD. In order to reveal new insights into the etiology and pathophysiology of PD, a new strategy is provided for the early diagnosis and treatment of PD from the perspective of the intestinal tract by targeting the gut microbiome.

## 2. The body-first and brain-first PD

Since Braak et al. discovered that pathological α-synuclein aggregated in the ENS may spread retrogradely to the brain through the vagus (19), a series of studies have shown that there is an important two-way interaction between the gut and the brain (20, 21). PD is assumed to exist in two subtypes: The brain-first PD, in which α-synuclein pathology spreads from the CNS affects the autonomic nervous system, and body-first PD, in which α-synuclein pathology originates from the intestinal or peripheral autonomic nervous system and then spreads to the CNS through vagus and sympathetic connections (22, 23). Compared to normal subjects, α-synuclein was found in the stomach and vagus of PD subjects (24). Human epidemiological data showed that complete truncal vagotomy could reduce the risk of secondary PD and delay the age of PD onset (25), indicating that α-synuclein is not only deposited in the substantia nigra but also in the gastrointestinal tract and the vagus. Animal studies have shown that α-synuclein aggregates can spread from the gastrointestinal tract to the brain through the autonomic nervous system. By injecting pathological α-synuclein into the duodenum and the pyloric muscle layer of mice (26) or the duodenal wall of rats (20), initial retrograde multi-synaptic propagation of pathological α-synuclein along with the loss of DN, motor and non-motor symptoms. However, trunk vagotomy and loss of α-synuclein could prevent the transmission of α-synuclein from the gut to the brain and the associated neurodegeneration and behavioral defects (26). Arotcarena et al. found that in non-human primate models, enteral injection or striatal injection of α-synuclein from PD patients can induce striatal injury and pathological manifestations of the ENS (21).

RBD is the strongest prodromal marker of PD, and clinical and imaging evidence suggests that RBD can be used as a clinical marker to distinguish body-first PD (RBD positive) from brain-first PD (RBD negative) (27, 28). The PET tracer 11C-Donepezil was used clinically to evaluate parasympathetic enteric innervation, and it was found that patients with RBD had reduced uptake of 11C-donepezil in the colon and small intestine (29), indicating that the body-first PD patients showed autonomic nerve innervation loss in the prodromal stage. This was verified in 37 newly diagnosed PD patients, using a multi-modal imaging case–control PET study which found that the 11C-donepezil intake of the colon in body-first type was significantly lower than that in the brain-first type (30). Cardiac 123I-MIBG imaging can effectively assess whether sympathetic dysfunction is present, and the study found that 92% of RBD patients show pathological 123I-MIBG imaging (27), and body-first PD patients show significantly a lower cardiac 123I-MIBG signal due to sympathetic dysfunction (30). The above imaging evidence supports the existence of brain-first and body-first subtypes of PD. In addition, colonic dysfunction can be quantified objectively by total colonic volume and colonic transit time (CTT). Studies have found that compared with healthy control groups, PD patients usually have significantly prolonged CTT and larger total colon volume (30, 31), among which total colon volume and CTT increase more significantly in patients with body-first PD (27).

Both clinical studies and animal model evidence (19–32) indicate that the dysfunctional autonomic nervous system (such as vagus) may be the pathway of pathological transmission of α-synuclein in PD between ENS and CNS, which is consistent with the body-first and brain-first hypothesis mechanism of PD.

## 3. Microbiota–gut–brain axis and intestinal microbiome dysregulation in PD

The gut microbiota is the densest microbiome in the human body, composed of bacteria, viruses, protozoa, fungi, etc., and communicates bidirectional with the brain through the microbiota gut–brain axis, thus significantly affecting the intestinal barrier function, inflammatory response, and nervous system function of the host (33, 34). The structure and function of the intestinal microbiome are constantly undergoing dynamic changes, which will be significantly affected by genetic factors and environmental factors (infection, medication, food, etc.), and the abnormal quantity or quality is called intestinal microbiota disorder (35). The dysregulation of intestinal microbiome in PD patients leads to increased exposure to various pro-inflammatory and neurotoxic microorganisms, and the changes in the entire intestinal microbiome are shown as a decreased level of short-chain fatty acids (SCFAs) and increased lipopolysaccharide (LPS) (36–38). In addition, intestinal microbiota can produce functional amyloid protein, namely, microbial amyloid protein, which can not only promote intestinal and systemic inflammation but also accelerate the aggregation of α-synuclein in the intestinal nerve plexus and spreads to the CNS through a transsynaptic cell-to-cell transmission (39). The above mechanisms may cause neuronal damage or promote susceptibility to neuronal damage, thus affecting the occurrence and development of PD (40).

### 3.1. Changes of major intestinal microbiota in PD and their correlation with clinical characteristics

The composition and function of intestinal microbiota are closely related to clinical characteristics of PD, including clinical symptoms, disease progression, and severity (41, 42). The high-throughput sequencing studies found that intestinal microbiome changes in PD patients persisted in follow-up sampling 2 years later (43), and the most significant changes were the decrease of the bacterial group producing SCFAs (with anti-inflammatory effects) and the increase of the bacterial group producing LPS (with pro-inflammatory properties) (44). With the development of PD, the abundance of *Faecalibacterium*, Roseburia, Prevotella, Lachnospiraceae family and their key member Butyrivibrio decrease significantly, while the abundance of Megasphaera, Akkermansia, and Verrucomicrobia as well as Lactobacillaceae continued to increase in PD patients (45–47). Among them, Roseburia decomposed carbohydrates to produce SCFAs, which can protect the gut from pathogens. The decreased abundance of Roseburia affects the host’s ability to repair epithelial cells and regulate inflammation and is associated with the deterioration of cognitive function. Prevotella decomposes proteins and carbohydrates to produce SCFAs, the abundance of which is negatively correlated with disease severity. Its abundance is significantly decreased in rapidly progressing PD patients and is associated with the deterioration of cognitive function (48). Butyrivibrio abundance decline is correlated with poor motor function and motor complications (49). The accumulation of Akkermansia promotes intestinal mucous barrier damage and intestinal inflammation, leading to abnormal aggregation of α-synuclein in the intestine, and eventually leads to higher endotoxemia and systemic inflammation to promote the progress of neuropathology (46). Increased abundance of Megasphaera is associated with poor motor and cognitive function (50). At the same time, changes in the composition of intestinal microbiota can affect neurodegeneration through inflammatory response, the abundance of Bacteroides is correlated with the level of plasma TNF-α and the severity of motor symptoms (51). In addition, reduced abundance of the major producers of butyrate (including the genera Roseburia, Romboutsia, and Prevotella) was associated not only with worsening cognitive function but also with the severity of depressive symptoms in PD patients (52) (Table 1).

**Table 1:** Summary of altered intestinal microbiota in PD and their correlation with clinical symptoms.

Bacteria	Abundance	Function	Motor Symptom	Non-motor Symptom	References
Roseburia	↓	Produce SCFAs	-	Be associated with the deterioration of cognitive function; Be associated with depressive symptom	Mao et al. (48); Xie et al. (52)
Prevotella	↓	Produce SCFAs	-	Be associated with the deterioration of cognitive function; Be associated with depressive symptom	Mao et al. (48); Xie et al. (52)
Butyrivibrio	↓	Produce butyrate	Be correlated with poor motor function and motor complication	-	Toh et al. (49)
Romboutsia	↓	Produce butyrate	-	Be associated with worsening cognitive function and depressive symptom	Xie et al. (52)
Akkermansia	↑	Degrade intestinal mucin	-	Promote gastrointestinal dysfunction	Nishiwaki et al. (46); Cirstea et al. (121)
Megasphaera	↑	-	Be associated with poor motor function	Be associated with poor cognitive function	Vascellari et al. (50)
Bacteroides	↑	-	Be correlated with severity of motor symptom	-	Lin et al. (51)

↓ refers to decreased abundance and ↑ refers to increased abundance.

At present, there is heterogeneity in the results of studies on the changes in intestinal microbiota in PD, which may be due to differences in research methods, disease status, and population as well as confounding factors (49). In order to elucidate the significance of changes in gut microbiome in PD and assess its potential as biomarkers for risk, diagnosis, treatment, and prognosis of PD, future large-scale clinical studies could employ a cross-comparative multi-omics approach combined with clear patient criteria (including geographic regions, ethnicity, disease stage, and detailed phenotypes and genotyping) to provide a comprehensive understanding of how the gut microbiome and its metabolites interact with the host and influence the cause, symptoms, and progression of PD. At the same time, more rigorous experimental design and more advanced detection methods are needed to deeply analyze the dynamic evolution process of intestinal microbiota in PD patients and animal models.

### 3.2. Changes in metabolites derived from gut microbiome

Microbial metabolites can not only reflect the composition and function of intestinal microbiota but also are closely related to the progression of PD. Abnormal microbial metabolites are correlated with the pathology of α-synuclein and the activation of microglia cells, which can promote the neurodegeneration and movement disorders of PD animal models (53). Among them, SCFAs are the main metabolite of dietary fiber fermentation by intestinal microflora (including acetic acid, propionic acid, and butyric acid.), which plays a key role in maintaining the integrity of the colon epithelium, regulating immune response and intestinal permeability, as well as affecting brain function (54). The case–control study confirmed that the fecal microbiome and metabolome composition of PD patients were significantly different from that of the control group, and the fecal SCFAs level and the bacteria-producing level were both decreased, but the plasma SCFAs level increased (55, 56), which is associated with impairment of the gut–blood barrier and may be aggravated by constipation (57). Metagenomic functional analysis confirmed differences in microbiome metabolism related to SCFAs precursor metabolism in PD patients (48). Microbial metabolite levels related to the relative abundance of the proinflammatory intestinal microbes, in PD patients, and the abundance of proinflammatory microorganisms such as Clostridiales bacterium and Ruminococcus sp. is significantly correlated with the decrease of SCFAs level in feces and the increase of SCFAs level in plasma, especially propionic acid (58). SCFAs levels in feces and plasma of PD patients are not only correlated to specific changes in intestinal microbiome but also closely related to the clinical severity of PD (59). Specifically, poor cognitive function of PD patients was significantly correlated with low SCFAs level in feces (55), high butyric acid, and valerate level in plasma (58). The poorer the motor function, the lower the fecal SCFAs level, and the higher the plasma propionic acid concentration (58), and the poor postural instability–gait disorder score is associated with a low butyric acid level (55). Meanwhile, elevated microbial metabolites in the plasma of PD patients include indole derivatives, secondary bile acids, and hippuric acid (HA), which act as signaling molecules that can cross the blood–brain barrier to regulate inflammatory response and metabolic homeostasis. Among them, the plasma HA level is correlated with PD disease status (60). The elevated plasma levels of Trimethylamine N-oxide derived from gut microbes through dietary components, including L-carnitine and choline, are associated with disease severity and progression of PD (61) (Table 2). In addition, preclinical studies have found changes in intestinal microbiota and metabolites in various animal models of PD, and restoring healthy intestinal microbiota can effectively improve the damage of dopamine neurons in animal models of PD. MPTP-induced mouse models with a reduced abundance of Faecalicatena was accompanied by decreased expression of propionic acid and striatal Tyrosine hydroxylase (TH) (62). A fasting-simulated diet increases favorable gut microbiome and SCFAs in PD mice, thereby increasing brain-derived neurotrophic factor (BDNF) levels and reducing neuroinflammation (63). Osteocalcin can improve the dyskinesia and DN loss of PD mice by increasing Bacteroidetes and the level of propionic acid (64).

**Table 2:** Changes of microbial metabolites and their effects on PD.

Microbial metabolites	Function	Plasma level	Effect on PD	References
Short-chain fatty acids (SCFAs)	Maintain the integrity of the colon epithelium; Regulate immune response and intestinal permeability; Affect brain function	↑	Be related to abundance of proinflammatory intestinal microbes; Be related to poor cognitive function; Be related to poor motor function	Tan et al. (55); Nuzum et al. (56); Chen et al. (58); Wallen et al. (59);
Hippuric acid	Regulate the brain’s inflammatory response and metabolic homeostasis	↑	Be correlated with PD disease status	Chen et al. (60)
Trimethylamine N-oxide	Promote α-synuclein aggregations and neuroinflammation	↑	Be associated with disease severity and motor symptom progression	Chen et al. (61)

↓ refers to decreased plasma level and ↑ refers to increased plasma level.

The clinical correlation between intestinal microbes with their metabolites and PD further supports intestinal microbes as new biomarkers for early diagnosis of PD and potential targets for treatment. Moreover, changes in intestinal microbiota composition affect fecal metabolomics characteristics. Therefore, fecal metabolomics can be used to better understand the association between intestinal microbiota and clinical features (including clinical phenotype, disease status, and progression) in PD patients.

## 4. Genetic and environmental factors contribute to the microbiota-gut-brain axis disturbance

The interaction between genetic susceptibility and environmental factors jointly promotes the occurrence and development of PD (65). Studies have shown that >85% of PD cases occur in a sporadic manner, and familial PD can be attributed to disease-causing gene mutations associated with PARK sites, including Parkin, PINK1, and LRRK2. Epidemiological data indicated that less than 50% of LRRK2 mutation carriers eventually develop PD (66), suggesting that environmental factors other than genetic mutations are needed to trigger PD. Neuropathological studies have shown that α-synuclein can spread from ENS to central DA, and age is the key factor for the spread of α-synuclein. Inoculation of α-synuclein into the gastrointestinal tract of elderly rats, α-synuclein transmits along enteric nerve (67) or sympathetic and parasympathetic nerves (68) to the brain. Mitochondria are key participants in inducing, promoting, or aggravating the pathogenesis of PD (69). Mitochondrial damage is involved in the inflammatory cascade (70, 71). Intestinal microbial disorders in PD patients may lead to the progressive loss of DN through mitochondrial dysfunction (72, 73).

The gastrointestinal tract is an important place of contact with the environment, and environmental risk factors related to PD, including infection, environmental pollutants, and pressure, can affect the intestinal microbiome, which is the trigger for the occurrence and development of PD in genetically susceptible hosts (74). A prospective cohort study involving 228,485 individuals aged 50 and above found that gastrointestinal infection was associated with an increased risk of PD, and the destruction of the gastrointestinal mucosa by bacterial and viral pathogens could trigger the aggregation of α-synuclein in intestinal neurons and initiate its retrograde transport to CNS (75). Repeated infection of intestinal *Citrobacter rodentium* can damage DN in PINK1^−/−^ mice and lead to motor deficiency (76). Further studies have revealed changes in intestinal microbiota over time, including the increased abundance of Enterobacteriaceae and Verrucomicrobia (77). The above studies have shown that differences in intestinal microflora caused by gastrointestinal infection can trigger PD. After long-term administration of rotenone, α-synuclein accumulation was observed in the CNS and intestine of mice (78), and the development of motor dysfunction depend on the presence of intestinal microbiota, compared with sterile mice, the changes in intestinal microbiota composition in conventionally fed mice were the same as those in human PD patients, including increased Lactobacillaceae, and decreased Lachnospiraceae (79). Chronic stress causes hypothalamic–pituitary–adrenal dysfunction in PD mice, leading to intestinal barrier dysfunction and decreased anti-inflammatory bacteria Lactobacillus abundance (80). The ingestion of trichloroethylene in elderly rats induces reduced abundance of Blautia that produced SCFAs (81).

The above research results indicated that the diversity and stability of intestinal microbiota decrease with age can lead to an increased genetic susceptibility to PD-related neurodegeneration, and environmental factors are more likely to trigger the pathophysiological process of PD microbe-gut-brain axis disorder.

## 5. Intestinal microbiome dysregulation and intestinal inflammation

The dysbiosis of intestinal microbiota can lead to intestinal inflammation, which can initiate the accumulation of misfolded α-synuclein in ENS in the early stage, and activate microglia and astrocytes through the microbiota-gut-brain axis upward pathway, thus triggering and/or promoting CNS inflammation and neurodegeneration. The above mechanism can have a synergistic effect with genetic and environmental factors to jointly trigger and promote the occurrence and development of PD (82, 83). Rota et al. found that in α-synuclein transgenic mice, significant symptoms of gastrointestinal dysfunction (such as constipation) precede CNS neurodegeneration (84). Further studies showed that the aggregation of α-synuclein in the colon of early PD mouse could trigger intestinal inflammation and induce impairment of the intestinal barrier, accompanied by reduced production of SCFAs such as butyric acid and propionic acid (85). Through the double-hit PD model, it was found that intestinal inflammation and microbial dysbiosis could promote mucosal barrier leakage, enhance intestinal inflammation in mice, and accompany DN loss (86, 87).

As producers of Toll-like sensors (TLRs) ligands, the dysregulation of intestinal microbiota causes damage to intestinal epithelial cells through the activation of TLRs, then triggered the downstream TLR4 signaling pathway, thus promoting the inflammatory response in the gut and brain of PD patients (88). Intestinal inflammation, neuroinflammation, intestinal dysfunction, and neurodegeneration were significantly reduced in PD rodent models with TLR4 knockout (89, 90). Some variants of the TLR4 genes are considered to be risk factors of inflammatory bowel disease (IBD) and PD (91). Intestinal inflammation, a hallmark of IBD, plays an important role in the occurrence and development of PD. Clinical studies have indicated that clear genetic and pathophysiological links between IBD and PD (92, 93), and IBD may moderately increase PD risk (94). Both IBD and PD have intestinal inflammation, intestinal barrier dysfunction, and intestinal microbiome dysbiosis (95). Dysregulation of intestinal microbiome is closely associated with chronic intestinal inflammation in IBD, IBD patients and PD patients had the same intestinal microbiome characteristics, showing pro-inflammatory microbiota profiles, with a lower microbial α-diversity，and the abnormal expression of α-synuclein has been found in both intestines and ENS of IBD patients (96, 97). Similar to the PD, the abundance of bacteria-producing SCFAs like Lachnospiraceae, Roseburia, Faecalibacterium, Ruminococcus and Blautia in patients with IBD decreased significantly (98), gut microbiota dysbiosis promotes the onset of IBD. Meanwhile, multiple cohort studies (99–102) and two systematic reviews and meta-analysis (103, 104) have found that irritable bowel syndrome (IBS) is associated with a higher hazard of PD. IBS is a functional bowel disorder characterized by recurrent abdominal pain and changes in bowel habits (105, 106). It has been found that intestinal inflammation, increased intestinal permeability and changes in intestinal microbiome are involved in the pathogenesis of IBS, which was similar to that of PD (107). A nested case–control study with 1.7 million participants suggested that IBS is associated with a higher risk of PD and support the importance of the microbiota-gut-brain axis in PD etiology (108). The above studies indicate that intestinal microbiome dysregulation promotes intestinal inflammation, which plays an important role in the pathogenesis of PD.

## 6. Intestinal microbiome dysregulation and gastrointestinal dysfunction

Clinical and neuropathological evidence shows that the neurodegeneration of PD is accompanied by gastrointestinal dysfunction (109–112). A retrospective study involving 1.5 million participants showed that the earliest estimated time of onset of PD prodromal gastrointestinal dysfunction occurred decades before motor symptoms (109). Heinzel et al. conducted a study on 666 elderly people and found that intestinal microbiota composition was related to PD precursor markers, and its changes would lead to changes in clinical symptoms (110).

### 6.1. Constipation

Constipation is the most common PD-related gastrointestinal dysfunction, which is considered as reliable evidence of autonomic nervous disorder in the PD prodromal stage (113). The severity of constipation can predict the progress of motor symptoms and cognitive impairment in PD patients and seriously affect their quality of life (114). Lubomski et al. found that PD patients were three times more likely to be constipated than healthy subjects (78.6 vs. 28.4%); age, stage, depression, anxiety, and autonomic dysfunction all increased the risk of constipation in PD patients (115); and the significantly reduced physical activity in PD patients was correlated with the severity of constipation (116). With the progression of the disease, the incidence of constipation in PD patients increases, and more than 80% of PD patients (including newly diagnosed PD patients) show prolonged CTT (117). At the same time, chronic constipation leads to slower gastrointestinal emptying, which can delay PD drug absorption (impaired pharmacodynamics) and thus lead to deterioration of motor function (118, 119). Clinical studies have proved that intestinal microbial dysregulation is related to gastrointestinal dysfunction in PD patients. According to the 16SrRNA gene sequence data of 324 participants, the effect of constipation on PD is as high as 76.56% mediated by intestinal microbial changes (120), and intestinal microflora dysbiosis plays an important role in PD-related constipation mainly through the reduction of SCFAs producing bacteria. Constipated PD patients show unique intestinal microbiota characteristics, namely, decreased butyrate synthesis, increased production of harmful amino acid metabolites, including an increase in Akkermansia and Bifidobacterium while a decrease in Faecalibacterium and Lachnospiraceae. Akkermansia was positively correlated with chronic constipation and stool hardness, while Faecalibacterium and bacteria-producing butyrate are negatively correlated with stool hardness and constipation (121). The above studies indicated that intestinal microbiota composition and metabolic changes in PD patients are closely related to intestinal function, and supplementation of probiotics containing SCFAs producing bacteria or drugs promoting the growth of SCFAs-producing bacteria which may have a potential application prospect in the prevention and treatment of PD-related constipation.

### 6.2. Small intestinal bacterial overgrowth (SIBO)

SIBO refers to a large amount of colonization of the small intestine by bacteria present in the colon (122). A meta-analysis involving 973 participants showed an increased prevalence of SIBO in PD patients (33–52%) and a strong association with motor complications (123). SIBO-positive patients exhibit increased intestinal permeability, bacterial translocation, promoting microglial cell activation and abnormal accumulation of α-synuclein in intestinal neurons, as well as affecting levodopa bioavailability due to peripheral inflammation or partial metabolism of levodopa. Van et al. found that PD patients with SIBO positive had a higher relative abundance of bacterial tyrosine decarboxylase in the proximal small intestine (the site of levodopa absorption), which reduces the level of levodopa *in situ* (10). Among the bacteria species identified so far, *Enterococcus faecalis* rich in tyrosine decarboxylase can fully metabolize levodopa peripheral (11). Meanwhile, PD treatment drugs may be an important confounder of intestinal microbiome changes, and dopamine agonists can cause SIBO in healthy rats, including an increase in Lactobacillus, and affect L-dopa bioavailability (17). The above studies have shown a negative correlation between bacteria with tyrosine decarboxylase activity and the level of levodopa in the systemic circulation, and PD-related drugs essentially have significant effects on disease-related complications, including promoting gastrointestinal dysfunction, SIBO, and altering intestinal microbiome composition (18). Therefore, specific bacterial species in the small intestine such as *Enterococcus faecalis*, Lactobacillus species, and tyrosine decarboxylase activity levels can be used as biomarkers to monitor the efficacy of levodopa. Future studies need to consider the effects of PD therapeutics and SIBO eradication on gastrointestinal motor function and microbiome composition.

### 6.3. *Helicobacter pylori* (HP) infection

HP infection has been found to be associated with the pathophysiology and increased risk of PD (124). Colonization of HP in the gastrointestinal tract leads to the destruction of the blood–brain barrier, neuroinflammation, and degradation of DN through direct neurotoxic effects (neurotoxic factors directly damage cells), local effects (chronic mucosal inflammation damages the gastrointestinal barrier), and systemic immune responses (increased secretion of pro-inflammatory cytokines). Meanwhile, HP induces the reduction of gastric acid that leads to dysregulation of the gut microbiome, contributing to the development of SIBO, as previously described, which worsens the motor function of PD (125). The retrospective cohort study found that compared with the control group (*n* = 9,105), the HP infection group (*n* = 9,105) had a significantly higher risk of PD (126). Another case–control study found that HP-positive patients had worse motor function (127). A meta-analysis of 13 studies showed that HP infection was associated with more severe motor symptoms and worse drug response in PD patients (12). Another meta-analysis of 10 studies found that the eradication of HP could improve motor retardation and myotonia in PD patients as well as improve the therapeutic outcome of levodopa (13). Clinical observation suggested that duodenal inflammation induced by HP infection is accompanied by mucosal damage, which leads to poor drug response and motion fluctuation in PD patients through impaired levodopa bioavailability (14). The above studies emphasize that HP infection is involved in the pathophysiological process of PD, which can not only worsen the severity of the disease but also negatively affect the drug response of patients. HP eradication may improve its bioavailability by reducing HP-dependent levodopa consumption, thus improving motor control (15). Considering the high clinical prevalence of HP infection, it may be reasonable to screen people with a high risk of PD for HP. Meanwhile, for PD patients with poor symptom control, HP eradication may enhance the effect of levodopa, but whether HP eradication affects the natural process or progression of PD remains to be verified by further large-scale longitudinal studies and randomized controlled trials.

The prodromal stage is a window of opportunity for better understanding the pathogenesis of PD and early detection of the disease. Gastrointestinal dysfunction is the most important non-motor symptoms in PD patients (128). Currently, the management and treatment of PD-related gastrointestinal dysfunction are limited (129). Studies have shown that not only levodopa and other therapeutic drugs can directly affect the microbiome but also the intestinal microbiome can interfere with the absorption and utilization of drugs. Therefore, it is crucial to identify and treat PD-related gastrointestinal dysfunction, and further studies are needed on the potential interactions between intestinal microbiota and therapeutic drugs used, so as to improve the bioavailability of drugs such as levodopa and provide a basis for the development of new complementary therapeutic strategies for PD at the intestinal level.

## 7. PD therapy: disease remission strategies based on regulation of the gut microbiome

Considering the role of the microbiota-gut-brain axis in the occurrence and progression of PD, disease mitigation strategies based on intestinal microbiome regulation deserve further research, especially in the prevention and treatment of gastrointestinal dysfunction and motor symptoms in PD. At present, preclinical and clinical studies mainly focus on reducing the clinical symptoms of PD or delaying the progression of the disease through probiotics, prebiotics, and diet adjustment (130).

### 7.1. Food and diet pattern

Diet and nutrition are the main factors affecting the balance of intestinal microbiota (131). Epidemiological reports showed that the regulation of intestinal microbiota through food and diet pattern can not only reduce the risk of PD (132) but also improve the symptoms and quality of life of PD patients (133, 134). There is a strong correlation between the age of PD onset and dietary habits, with adherence to the Mediterranean diet that can reduce the probability of precursor PD in the elderly (135). Adherence to the MIND diet is closely associated with delayed onset of PD in women, with the longest delay of 17.4 years, and adherence to the Greek Mediterranean diet is associated with delayed onset of PD in men, with a difference of up to 8.4 years (136). A negative association between Mediterranean diet adherence and PD was observed in a cohort of more than 47,000 Swedish women (137). Evidence from a systematic review involving 52 studies suggests that following a Mediterranean diet can reduce the onset and clinical progression of PD (138). Specific dietary patterns can regulate intestinal inflammation and influence the risk of PD (139). Western diet rich in refined carbohydrates and animal saturated fats, may have a harmful effect on the microbiota-gut-brain axis, which can lead to intestinal microbiome dysbiosis and increase bacteria containing a large amount of LPS, thus affecting intestinal barrier function and leading to endotoxemia, systemic inflammation, and mitochondrial dysfunction (140), which is associated with increased risk and deterioration of PD. Rich in flavonoids, polyunsaturated fatty acids, and plant fiber, the Mediterranean diet has a positive effect on the gut microbiome, which can increase SCFAs-producing bacteria and induce GLP-1 and BDNF release, reduce intestinal inflammation, and prevent neurodegeneration, thereby reducing the risk of PD (141). A case–control study with 54 PD patients showed that a vegetarian diet including a high proportion of anti-inflammatory acting short-fatty acids (SCFA) can improve the pro-inflammatory intestinal microbiome in PD patients, with a significant clinical improvement as quantified by UPDRS III (142). In addition, α-synuclein in food, which share cross-reaction epitopes with human α-synuclein and have molecular similarity with brain antigens were involved in synaptic nucleoprotein lesions in the pathogenesis of PD through autoimmunity (143, 144), including forming immune complexes with antibodies to cross the blood–brain barrier and also reaching the blood–brain barrier from ENS (145). Therefore, elimination of foods containing α-synuclein in the diet may help to prevent or delay the occurrence and development of PD (Table 3).

**Table 3:** Summary of the role of food and diet pattern in PD.

Food or Diet Pattern	Effect on PD	References
Mediterranean diet	Reduce the risk of PD	Maraki et al. (135); Yin et al. (137); Bianchi et al. (138); Bianchi et al. (141)
MIND diet	Delay the onset of PD in women	Metcalfe-Roach et al. (136)
Greek Mediterranean diet	Delay the onset of PD in men	Metcalfe-Roach et al. (136)
Western diet	Increase risk and deterioration of PD	Terenzi et al. (139); Jackson et al. (140);
Vegetarian diet	Improve clinical motor symptoms of PD	Hegelmaier et al. (142)
Containing α-synuclein in the diet	Be involved in the pathogenesis of PD through autoimmunity.	Vojdani et al. (143); Vojdani et al. (144); Lerner et al. (145)

The food and diet pattern may affect the microbiota-gut-brain axis by altering the composition of the microbiome, thereby improving the progression of PD. In future, it is necessary to further determine the potential beneficial effects of various dietary patterns in inhibiting amyloid accumulation and oxidative stress in ENS and better understand the effects of diet and intestinal microbial disorders on PD, including disease progression, autonomic dysfunction, and cognitive function. At the same time, the long-term nature of food and diet pattern needs to be considered, as well as the duration, dose, and combination of interventions for different dietary patterns.

### 7.2. Probiotics

Probiotics are live microorganisms that are beneficial to the health of the host when given in appropriate amounts, and preclinical and clinical studies have shown that probiotics regulate gut microbiota (improving intestinal barrier integrity, reducing overgrowth of potentially pathogenic bacteria in the gut, and inhibiting bacterial translocation), maintain immune homeostasis (regulating the immune system of the gastrointestinal mucosa), protect DN (inhibiting glial cell activation, increasing BDNF and SCFAs, and reducing LPS), and improve the overall PD behavioral phenotype (146). Intake of probiotics can not only improve constipation-related non-motor symptoms in PD patients but also alleviate motor dysfunction (147, 148). Multiple randomized controlled clinical trials have shown that the ingestion of probiotics (with multiple strains of probiotics alone (149, 150) or in combination with probiotic fibers (151)) can improve gastrointestinal symptoms in PD patients by modulating the microbiota-gut-brain axis, including reducing abdominal pain, bloating, and constipation symptoms, and improving stool hardness, bowel frequency, and bowel habits in PD patients with constipation (152). Therefore, probiotics relieve constipation by regulating intestinal microbiota, which has a good clinical application value (153). In addition, taking probiotics for 12 weeks can reduce MDS-UPDRS scores and improve insulin resistance in PD patients (154). At the same time, preclinical studies have found that probiotics can alleviate movement disorders in PD animal models and exert neuroprotective effects on DN. Long-term administration of probiotics can not only improve gastrointestinal symptoms and UPDRS scores of MitoPark PD mice but also inhibit the progressive degeneration of DN in the nigra (155). Goya et al. found that the probiotic *Bacillus subtilis* PXN21 could affect the release of intestinal microbial metabolites in *Caenorhabditis elegans*, thereby inhibiting and reversing the aggregation of α-synuclein and removing formed synuclein lesions (156). Intestinal microbiota can also affect the progression of PD by regulating intestinal endocrine through GLP-1, relieve oxidative stress and inflammatory response, and inhibit TH neuron apoptosis through activating its receptor GLP-1R (157). Ingestion of probiotics can reduce the intestinal pathogen Enterobacteriaceae in MPTP-induced PD mice (158), reverse the dysbiosis of intestinal microbiome (increased abundance of Alistipes) (159), and increase TH-positive neurons by increasing GLP-1.

Considering the high variability of the inherent intestinal microbiota from PD patients and exogenous probiotics, a further longitudinal study is needed on the influence of exogenous probiotics on the intestinal microenvironment of PD patients before and after intervention under optimal control conditions and to verify the long-term efficacy, safety, and mechanism of its treatment of PD. Meanwhile, accurate development of personalized treatment plans requires the determination of the most appropriate probiotics for PD treatment based on the specific intestinal microbiota profile of a single PD patient.

## 8. Conclusion

In summary, the preclinical and clinical research evidence discussed in this review supports the important role of bidirectional microbiota-gut-brain pathways and intestinal microbiome dysregulation in the initiation and progression of PD. In the condition of intestinal microbiota dysbiosis, the pro-inflammatory microenvironment may induce α-synuclein deposited in ENS to spread to the CNS in the form of transsynaptic cell transmission and further causes gastrointestinal dysfunction, neuroinflammation, and neurodegeneration through the disordered microbiota-gut-brain axis. The relationship between intestinal microbiota disorder and PD is far more complex than the one-way causal relationship. Elucidating the pathophysiological role of the microbiota-gut-brain axis in PD can not only further reveal the early pathogenesis of PD and predict the progression of neurodegeneration, phenotypic transformation, and prognosis but also intestinal microbiome-oriented treatment strategies to maintain or restore the homeostasis of the intestinal microenvironment may alter the disease course of PD through the microbiota-gut-brain axis, which will provide future direction for the development of new biomarkers for early diagnosis and therapeutic targets to slow the progression of PD. This can be applied clinically to design more effective personalized or subtype-specific, patient-centered treatment and prevention strategies.

## Author contributions

QL, L-bM, and Q-yY contributed to conception of the review. QL and L-bM wrote the first draft of the manuscript. L-jC, XS, LT, QZ, J-lY, XL, and YZ wrote sections of the manuscript. Q-yY was involved in critically revising the manuscript for important intellectual content. All authors contributed to the article and approved the submitted version.

## Conflict of interest

The authors declare that the research was conducted in the absence of any commercial or financial relationships that could be construed as a potential conflict of interest.

## Publisher’s note

All claims expressed in this article are solely those of the authors and do not necessarily represent those of their affiliated organizations, or those of the publisher, the editors and the reviewers. Any product that may be evaluated in this article, or claim that may be made by its manufacturer, is not guaranteed or endorsed by the publisher.
